# The different maturation of the corticospinal tract and corticoreticular pathway in normal brain development: diffusion tensor imaging study

**DOI:** 10.3389/fnhum.2014.00573

**Published:** 2014-08-04

**Authors:** Sang Seok Yeo, Sung Ho Jang, Su Min Son

**Affiliations:** ^1^Department of Physical Therapy, College of Health Sciences, Dankook UniversityCheonan, South Korea; ^2^Department of Physical Medicine and Rehabilitation, College of Medicine, Yeungnam UniversityTaegu, South Korea

**Keywords:** corticospinal tract, corticoreticular pathway, maturation, motor, brain

## Abstract

**Background and Purpose:** The corticospinal tract (CST) and corticoreticular pathway (CRP) are known to be important neural tracts for motor development. However, little is known about the difference in maturation of the CST and CRP. In this study, using diffusion tensor imaging (DTI), we investigated maturation of the CST and CRP in typically developed children and normal healthy adults.

**Methods:** We recruited 75 normal healthy subjects for this study. DTI was performed using 1.5-T, and the CST and CRP were reconstructed using DTI-Studio software. Values of fractional anisotropy (FA) and fiber volume (FV) of the CST and CRP were measured.

**Results:** In the current study, the threshold points for CST and CRP maturation were different in normal brain development. Change in FA value of the CST showed a steep increase until 7 years of age and then a gradual increase until adulthood, however, the CRP showed a steep increase only until 2 years of age and then a very gradual increase or plateau until adulthood. In terms of FV, the CST showed a steep increase until 12 years and then a gradual increase until adulthood, in contrast, the CRP showed gradual increase of FV across whole age range (0–25 years).

**Conclusion:** The difference in maturation process between CST and CRP appears to be related to different periods of fine and gross motor development. This radiologic information can provide a scientific basis for understanding development in motor function.

## Introduction

Motor development in humans is achieved by functional use of trunk and limb muscles (Sutherland et al., 1980; Molnar, 1992; Pollak, 1993; Hausdorff et al., 1999; Williams and Monsma, 2006; Mayson et al., 2007; Hadders-Algra, 2010; Gouelle et al., 2011; Wu et al., 2011; Froehle et al., 2013). In detail, this motor development is generally divided into two different types of motor skills, gross motor skills and fine motor skills (Molnar, 1992; Pollak, 1993). Gross motor skills mainly require the use of proximal and axial muscles for postural control and locomotion (Sutherland et al., 1980; Hausdorff et al., 1999; Williams and Monsma, 2006; Mayson et al., 2007; Gouelle et al., 2011; Wu et al., 2011; Froehle et al., 2013), while fine motor skills require more precise movements, such as functional use of hands (Molnar, 1992; Pollak, 1993; Savion-Lemieux et al., 2009; Hadders-Algra, 2010; Timmons et al., 2012).

These motor functions are related to the descending motor pathways classified as the corticospinal tract (CST, pyramidal tract) and the non-CST (extra-pyramidal tract). The CST is known to be primarily involved in fine motor skills, such as hand function (York, 1987; Ahn et al., 2006; Schaechter et al., 2009; Lo et al., 2010; Wang et al., 2010). On the contrary, the corticoreticular pathway (CRP), one of the extrapyramidal motor pathways, is known to be concerned with innervation of the proximal and axial muscles involved in gross motor skills, such as postural control and locomotion (Matsuyama et al., 2004; Yeo et al., 2012a,b, 2013; Do et al., 2013). These different functional roles of the CST and CRP in motor control have been reported in many previous studies using diffusion tensor imaging (DTI; Ahn et al., 2006; Schaechter et al., 2009; Yeo et al., 2012b, 2013; Do et al., 2013; Jang et al., 2013).

DTI is a recently introduced technique that enables estimation of the integrity of the white matter tract by virtue of its ability to visualize the diffusion characteristics of water (Mori et al., 1999). Diffusion tensor tractography (DTT), a three-dimensional visualized version of DTI, provides a concrete description of the architecture and integrity of the CST and CRP (Ahn et al., 2006; Schaechter et al., 2009; Yeo et al., 2012a,b, 2013; Do et al., 2013; Jang et al., 2013). Several previous studies have demonstrated that DTI and DTT are very helpful in estimation of the state of neural tracts (Mori et al., 1999; Kunimatsu et al., 2004; Smith et al., 2004; Yeo et al., 2012b, 2013; Do et al., 2013; Jang et al., 2013). However, little is known about differences in maturation of the CST and CRP (Lebel and Beaulieu, 2011).

In the current study, using DTI, we attempted to investigate maturation of the CST and CRP in typically developed children and normal healthy adults.

## Materials and methods

### Subjects

**Figure 1:**
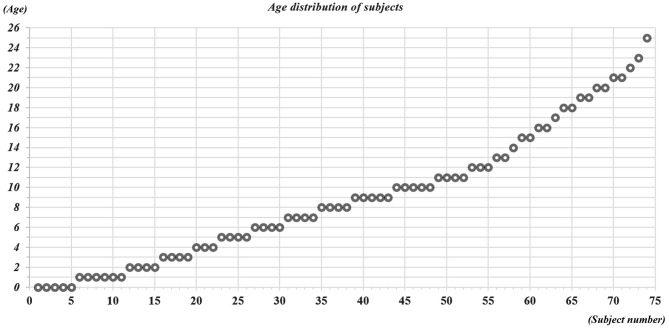
**Age distribution of subjects between 0–25 years old**.

A total of 75 normal healthy subjects (43 males, 32 females; mean age, 9.7 ± 6.6 years; range, 3 months to 25 years) with no history of neurologic disease or brain trauma were recruited for this study (Figure 1). All participants underwent evaluation by a neurologist and were diagnosed as normal healthy subjects. All participants or parents of participants provided written informed consent and the study was approved by the institutional review board of a university hospital.

### Diffusion tensor imaging

DTI data were acquired using a 1.5-T Philips Gyroscan Intera system equipped with a synergy-L Sensitivity Encoding (SENSE) head coil using a single-shot, spin-echo planar imaging pulse sequence. For each of the 32 noncollinear and noncoplanar diffusion sensitizing gradients, we acquired 60 contiguous slices parallel to the anterior commissure-posterior commissure line. The imaging parameters were: matrix = 128 × 128 matrix, field of view = 221 × 221 mm^2^, TE = 76 ms, TR = 10,726 ms, SENSE factor = 2; EPI factor = 67 and *b* = 1000 mm^2^s^−1^; NEX = 1; and a slice thickness of 2.3 mm.

**Figure 2:**
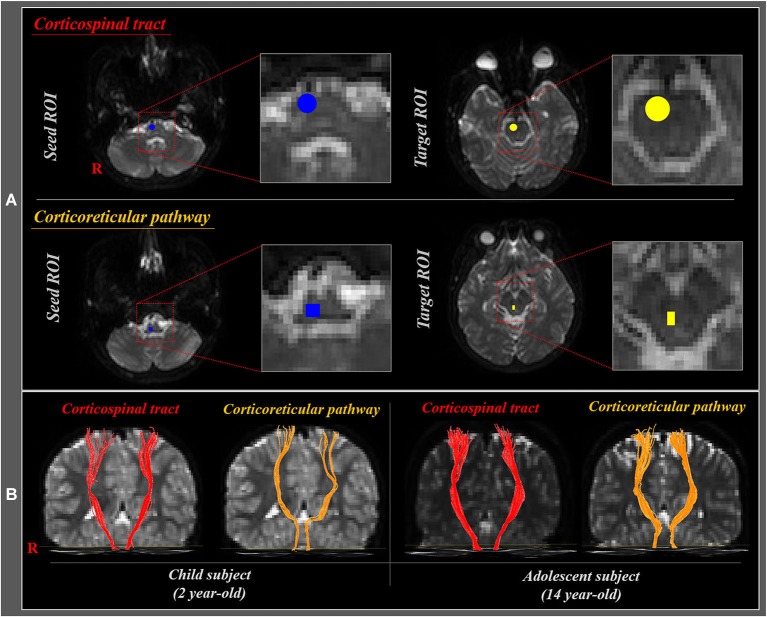
**(A)** Seed and target region of interest (ROI) of the corticospinal tract (CST) and corticoreticular pathway (CRP). For the CST, seed ROI was placed on the CST portion of the pontomedullary junction (blue circle), and the target ROI on the CST portion of the anterior mid-pons (yellow circle). For the CRP, seed ROI was given on the medullary reticular formation (blue rectangle), and the target ROI was placed on the midbrain tegmentum (yellow rectangle). **(B)** Diffusion tensor tractography (DTT) of the CST and CRP in child (2 year-old) and adolescent (14 year-old) subjects.

Affine multi-scale two-dimensional registration was used for reduction of eddy current-induced image distortions and motion artifacts (Smith et al., 2004). Preprocessing of DTI datasets was performed using the Oxford Centre for Functional Magnetic Resonance Imaging of Brain (FMRIB) Software Library (FSL). DTI-Studio software (CMRM, Johns Hopkins Medical Institute, USA) was used for reconstruction of the CST and CRP. For analysis of the CST, the seed region of interest (ROI) was placed on the CST portion of the pontomedullary junction, and the target ROI on the CST portion of the anterior mid-pons (Yeo et al., 2012b; Seo and Jang, 2013). For analysis of the CRP, the seed ROI was placed on the reticular formation of the medulla, and the target ROI on the midbrain tegmentum (Yeo et al., 2012a, 2013; Do et al., 2013; Jang et al., 2013; Figure 2A). The CST and CRP were determined by selection of fibers passing through seed and target ROIs (Figure 2B). Fiber tracking was performed using a fractional anisotropy (FA) threshold of >0.15 and direction threshold <70°. We measured the FA and fiber volume (FV) values of the CST and CRP in both hemispheres.

### Statistical analysis

Piecewise regression analysis was used for determination of the difference of maturation period in the CST and CRP according to age. Piecewise regression analysis is good to demonstrate a change in slope and possible break point when it occurs compared to the previous segment. Shifts in slope with *p* < 0.01 related to the studied reforms were considered as statistically significant. SAS software (v.15.0; SAS, SAS Inst. Inc. Cary, NC) was used in performance of statistical analyses.

## Results

**Figure 3:**
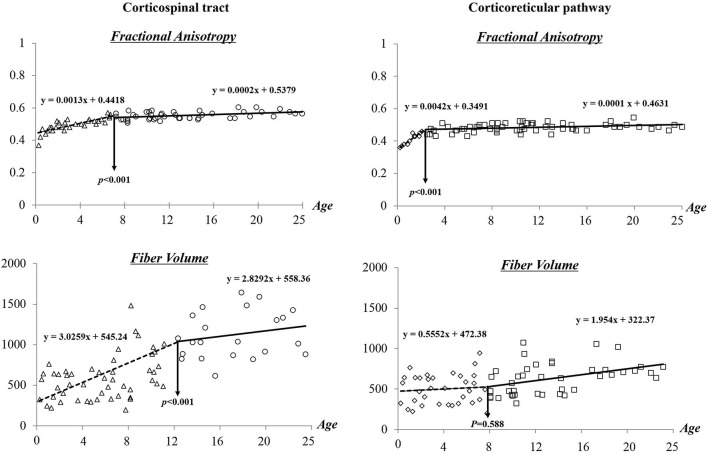
**Difference of maturation period in the corticospinal tract and corticoreticular pathway according to age**.

Figure 3 shows the results of segmented regression analysis for age-related change of FA and FV of the CST and CRP. From segmented regression lines, “threshold points”, where a minute age change (X axis) would make a different slope in the graph, that is, from a steep increase to a gradual increase or from a gradual increase to a steep increase in FA or FV (Y axis) could be found. This threshold point revealed a difference between FA and FV, and between CST and CRP. In detail, the FA value of the CST showed a progressive increase across the age range of 0–7 years (*y* = 0.0013*x* + 0.4418) (*R*^2^ = 0.762), and subsequent slow changes were observed across the range of 8–25 years old (*y* = 0.0002*x* + 0.5379). By contrast, the CRP showed a steep increase of FA value within the age range of 0–2 years (*y* = 0.0042*x* + 0.3491) (*R*^2^ = 0.625), and a subsequent leveling off in change of FA value was observed after 2 years of age (*y* = 0.0001*x* + 0.4631). In terms of FV, progressive increases in volumes of the CST were observed with age (0–25 years); more gradual increases in FV were observed at 12 years of age (0–11: *y* = 3.0259*x* + 545.24, 12–25: 2.8292*x* + 558.36) (*R*^2^ = 0.277). In contrast, CRP showed gradual increase of FV across whole age range (0–25 years) although there was insignificant change at 8 years old.

## Discussion

In the current study regarding the maturation of the CST and CRP, it was found that threshold points for CST and CRP maturation were different in normal brain development. Several studies have reported on maturation of the CST in normal development (Koh and Eyre, 1988; Nezu et al., 1997; Fietzek et al., 2000; Paus et al., 2001). In 1988, using electromagnetic stimulation, Koh and Eyre (1988) reported maturation of the CST in 142 in healthy subjects (from 33 weeks’ gestation to 50 years). They suggested the stepwise increment of sensitivity of the CST to electromagnetic stimulation between 8 and 11 years of age, and progressive increase of conduction velocity with increasing age; adult values are attained at about 11 years of age. In 1997, Nezu et al. (1997) demonstrated electromyographic responses of the CST to transcranial magnetic stimulation in normal children ranging in age from 1 to 14 years. They suggested that the maturity of the CST, which controls the intrinsic hand muscles, is electrophysiologically complete at 13 years of age. In a recent study, using DTI, Lebel and Beaulieu (2011), who reported on the increment of tract volume in the CST across the age span of 5–32 years, demonstrated significant increment of FA value of the CST in normal children (5–11 years-old). However, they did not include older subjects, such as adolescents or young adults; significant change from childhood to the adolescent period has not been identified. Our results included from infants to adulthoods appeared to nearly coincide with those of the above mentioned previous studies. However, no study on maturation of the CRP in normal development has been reported.

The results of the current study also coincided with the known developmental progress of motor function in humans. In detail, fine motor skills of upper extremities are required for use of fingers, hands, and arms; these include movements of reaching, grasping, and manipulation of objects (Molnar, 1992; Pollak, 1993; Savion-Lemieux et al., 2009). At the age of 1 year, children can pinch and hold small objects using the thumb and index finger, and are able to transfer objects from one hand to the other hand voluntarily. At the age of 2–3 years, children can reach their arm while maintaining posture, and show more refined wrist and finger movements. At the age of 4–6 years, children show more developed hand function by decreased use of elbow and shoulder. Basic motor control of hand movements continues to mature until the age of 6–8 years (Molnar, 1992; Hadders-Algra, 2010; Timmons et al., 2012). However, development of more functional movement and precise fine motor skills, such as motor timing and sensorimotor integration, is known to continue until young adulthood (Savion-Lemieux et al., 2009). Our results regarding FA threshold point of the CST at 7 years might be related to acquisition of basic control of hand movements, such as grasping, reaching, and simple manipulation of objects; by contrast, continuous increment of FV can contribute to maturation of more precise fine motor skills, such as motor timing and motor integration. On the other hand, gross motor skills are required for control of the proximal muscles for locomotion or postural control (Sutherland et al., 1980; Hausdorff et al., 1999; Williams and Monsma, 2006; Mayson et al., 2007; Gouelle et al., 2011; Wu et al., 2011; Froehle et al., 2013). Acquisition of gait function usually occurs at the age of 12–15 months, however, children at these ages usually show a wide-based gait with hyperflexion of hips and knees (Sutherland et al., 1980; Molnar, 1992; Pollak, 1993), whereas, children at the age of 2 years show a more mature gait pattern with reciprocal arm swing and heel-strike with increased stride length and velocity (Sutherland et al., 1980). Officially, maturation to an adult-like pattern is known to be acquired at or around the age of 7 years (Hausdorff et al., 1999; Gouelle et al., 2011). However, spatiotemporal parameters of gait ability continue to mature during the ages of 8–18 years (Froehle et al., 2013); cadence, step length, base of support, and initial double support time continue to mature during adolescence. The authors suggest that steep increment of FA in the CRP until 2 years of age might be related to the first acquisition of gait function. By contrast, progressive increment of FV in the CRP across the whole age range might be related to continuous maturation of the gait function until adolescence.

In previous studies, FA value is known to represent the degree of completion for white matter organization; in detail, increased FA values indicate greater unidirectionality of well-organized white matter tracts, and decreased FA values reflect impaired or more immature microstructure of the white matter tracts (Mori et al., 1999; Rha et al., 2012). Many previous studies have reported significant correlation between decrease of FA value and poor motor function (Ahn et al., 2006; Schaechter et al., 2009; Yeo et al., 2012b; Jang et al., 2013). In addition, myelination of the white matter is a crucial component of neurologic development which concerned with the development of sensory, motor, and cognitive function in young children (Nagy et al., 2004; Drobyshevsky et al., 2005). Many previous studies suggested that the changes of diffusion parameters, the FA and mean diffusivity, closely related to the maturation process of the white matter myelination (Nagy et al., 2004; Drobyshevsky et al., 2005; Hüppi and Dubois, 2006; Welker and Patton, 2012). Compared with FA value, FV is determined by the number of voxels contained within a neural tract and is known as a quantitative measure of connectivity (Mori et al., 1999; Kwak et al., 2010; Rha et al., 2012). Therefore, progressive increment of FV in the neural tract indicates increased numbers of neural fibers (Mori et al., 1999; Kwak et al., 2010; Rha et al., 2012). In addition, some previous studies have reported that increment of FV in the CST and CRP was related to significant recovery of motor function after severe motor dysfunction caused by brain injury (Kwak et al., 2010; Jang et al., 2013). Our results regarding different threshold points of maturation of FA and FV in the CST and CRP coincided with those of these previous studies. The authors suppose that the maturation of FA, which is associated with completion of the neural tract organization, play an important role in the first acquisition of motor function in normal development, while the maturation of FV, representing quantity of connectivity, is related to development of higher functional skill.

In conclusion, according to our findings, FA value of the CST and CRP showed a steady increase with age and nearly adult like level skill was attained at the ages of 7 and 2 years, respectively. In addition, the FVs of the CST and CRP showed progressive increment with age. This is the first study to demonstrate differences in maturation between CST and CRP during normal motor development. However, limitations of this study should be considered. First, DTI may underestimate or overestimate the neural fiber tracts because regions of fiber complexity. Second, crossing fibers can prevent full reflection of the underlying fiber architecture. A recent previous study showed that crossing fibers can be detected in over 90% of white matter voxels (Jeurissen et al., 2010), and regions with complex crossing fiber tend to have lower FA values, compared with predominantly unidirectional white matter (Parker and Alexander, 2005; Yamada, 2009). Third, detailed and identical clinical data could not be obtained due to various distributions of age in subjects. Lastly, the major potential limitation of this study was the partial volume effect due to the same acquisition parameter for the DTI in all age. The partial volume effect can cause the underestimation of the FV in younger child subject compared with adult subject. Therefore, further analysis with partial volume correction, or alternatively with voxel based morphometric methods to more closely delineate maturation of the CST and CRP. In addition, combined study with TMS would also be necessary in order to compensate for this limitation of DTI. Future studies with improved imaging and modeling methods combined with neurological measures may further elucidate the subtle changes in maturation.

## Conflict of interest statement

The authors declare that the research was conducted in the absence of any commercial or financial relationships that could be construed as a potential conflict of interest.

## References

[B1] Ahn Y. H. Ahn S. H. Kim H. Hong J. H. Jang S. H. (2006). Can stroke patients walk after complete lateral corticospinal tract injury of the affected hemisphere? Neuroreport 17, 987–990 10.1097/01.wnr.0000220128.01597.e0 16791089

[B2] Do K. H. Yeo S. S. Lee J. Jang S. H. (2013). Injury of the corticoreticular pathway in patients with proximal weakness following cerebral infarct: diffusion tensor tractography study. Neurosci. Lett. 546, 21–25 10.1016/j.neulet.2013.04.040 23643994

[B3] Drobyshevsky A. Song S. K. Gamkrelidze G. Wyrwicz A. M. Derrick M. Meng F. (2005). Developmental changes in diffusion anisotropy coincide with immature oligodendrocyte progression and maturation of compound action potential. J. Neurosci. 25, 5988–5997 10.1523/jneurosci.4983-04.2005 15976088 PMC6724805

[B4] Fietzek U. M. Heinen F. Berweck S. Maute S. Hufschmidt A. Schulte-Monting J. (2000). Development of the corticospinal system and hand motor function: central conduction times and motor performance tests. Dev. Med. Child Neurol. 42, 220–227 10.1111/j.1469-8749.2000.tb00076.x 10795559

[B5] Froehle A. W. Nahhas R. W. Sherwood R. J. Duren D. L. (2013). Age-related changes in spatiotemporal characteristics of gait accompany ongoing lower limb linear growth in late childhood and early adolescence. Gait Posture 38, 14–19 10.1016/j.gaitpost.2012.10.005 23159678 PMC3580126

[B6] Gouelle A. Megrot F. Presedo A. Pennecot G. F. Yelnik A. (2011). Validity of functional ambulation performance score for the evaluation of spatiotemporal parameters of children’s gait. J. Mot. Behav. 43, 95–100 10.1080/00222895.2010.538768 21298587

[B7] Hadders-Algra M. (2010). Variation and variability: key words in human motor development. Phys. Ther. 90, 1823–1837 10.2522/ptj.20100006 20966209

[B8] Hausdorff J. M. Zemany L. Peng C. Goldberger A. L. (1999). Maturation of gait dynamics: stride-to-stride variability and its temporal organization in children. J. Appl. Physiol. (1985) 86, 1040–1047 10066721 10.1152/jappl.1999.86.3.1040

[B9] Hüppi P. S. Dubois J. (2006). Diffusion tensor imaging of brain development. Semin. Fetal. Neonatal. Med. 11, 489–497 10.1016/j.siny.2006.07.006 16962837

[B10] Jang S. H. Chang C. H. Lee J. Kim C. S. Seo J. P. Yeo S. S. (2013). Functional role of the corticoreticular pathway in chronic stroke patients. Stroke 44, 1099–1104 10.1161/STROKEAHA.111.000269 23444306

[B11] Jeurissen B. Leemans A. Tournier J. Jones D. K. Sijbers J. (2010). Estimating the number of fiber orientations in diffusion MRI voxels: a constrained spherical deconvolution study. Proc. Intl. Soc. Mag. Reson. Med. 18, 573

[B12] Koh T. H. Eyre J. A. (1988). Maturation of corticospinal tracts assessed by electromagnetic stimulation of the motor cortex. Arch. Dis. Child. 63, 1347–1352 10.1136/adc.63.11.1347 3202641 PMC1779172

[B13] Kunimatsu A. Aoki S. Masutani Y. Abe O. Hayashi N. Mori H. (2004). The optimal trackability threshold of fractional anisotropy for diffusion tensor tractography of the corticospinal tract. Magn. Reson. Med. Sci. 3, 11–17 10.2463/mrms.3.11 16093615

[B14] Kwak S. Y. Yeo S. S. Choi B. Y. Chang C. H. Jang S. H. (2010). Corticospinal tract change in the unaffected hemisphere at the early stage of intracerebral hemorrhage: a diffusion tensor tractography study. Eur. Neurol. 63, 149–153 10.1159/000281108 20134168

[B15] Lebel C. Beaulieu C. (2011). Longitudinal development of human brain wiring continues from childhood into adulthood. J. Neurosci. 31, 10937–10947 10.1523/JNEUROSCI.5302-10.2011 21795544 PMC6623097

[B16] Lo R. Gitelman D. Levy R. Hulvershorn J. Parrish T. (2010). Identification of critical areas for motor function recovery in chronic stroke subjects using voxel-based lesion symptom mapping. Neuroimage 49, 9–18 10.1016/j.neuroimage.2009.08.044 19716427

[B17] Matsuyama K. Mori F. Nakajima K. Drew T. Aoki M. Mori S. (2004). Locomotor role of the corticoreticular-reticulospinal-spinal interneuronal system. Prog. Brain Res. 143, 239–249 10.1016/s0079-6123(03)43024-0 14653169

[B18] Mayson T. A. Harris S. R. Bachman C. L. (2007). Gross motor development of Asian and European children on four motor assessments: a literature review. Pediatr. Phys. Ther. 19, 148–153 10.1097/pep.0b013e31804a57c1 17505292

[B19] Molnar G. E. (1992). Pediatric Rehabilitation. Baltimore: Williams and Wilkins

[B20] Mori S. Crain B. J. Chacko V. P. van Zijl P. C. (1999). Three-dimensional tracking of axonal projections in the brain by magnetic resonance imaging. Ann. Neurol. 45, 265–269 10.1002/1531-8249(199902)45:2<265::aid-ana21>3.0.co;2-3 9989633

[B21] Nagy Z. Westerberg H. Klingberg T. (2004). Maturation of white matter is associated with the development of cognitive functions during childhood. J. Cogn. Neurosci. 16, 1227–1233 10.1162/0898929041920441 15453975

[B22] Nezu A. Kimura S. Uehara S. Kobayashi T. Tanaka M. Saito K. (1997). Magnetic stimulation of motor cortex in children: maturity of corticospinal pathway and problem of clinical application. Brain Dev. 19, 176–180 10.1016/s0387-7604(96)00552-9 9134188

[B23] Parker G. J. Alexander D. C. (2005). Probabilistic anatomical connectivity derived from the microscopic persistent angular structure of cerebral tissue. Philos. Trans. R. Soc. Lond. B Biol. Sci. 360, 893–902 10.1098/rstb.2005.1639 16087434 PMC1854923

[B24] Paus T. Collins D. L. Evans A. C. Leonard G. Pike B. Zijdenbos A. (2001). Maturation of white matter in the human brain: a review of magnetic resonance studies. Brain Res. Bull. 54, 255–266 10.1016/s0361-9230(00)00434-2 11287130

[B25] Pollak M. (1993). Textbook of Developmental Pediatrics. Singapore: Churchill Livingstone

[B26] Rha D. W. Chang W. H. Kim J. Sim E. G. Park E. S. (2012). Comparing quantitative tractography metrics of motor and sensory pathways in children with periventricular leukomalacia and different levels of gross motor function. Neuroradiology 54, 615–621 10.1007/s00234-011-0996-2 22170081

[B27] Savion-Lemieux T. Bailey J. A. Penhune V. B. (2009). Developmental contributions to motor sequence learning. Exp. Brain Res. 195, 293–306 10.1007/s00221-009-1786-5 19363605

[B28] Schaechter J. D. Fricker Z. P. Perdue K. L. Helmer K. G. Vangel M. G. Greve D. N. (2009). Microstructural status of ipsilesional and contralesional corticospinal tract correlates with motor skill in chronic stroke patients. Hum. Brain Mapp. 30, 3461–3474 10.1002/hbm.20770 19370766 PMC2780023

[B29] Seo J. P. Jang S. H. (2013). Characteristics of corticospinal tract area according to pontine level. Yonsei Med. J. 54, 785–787 10.3349/ymj.2013.54.3.785 23549830 PMC3635640

[B30] Smith S. M. Jenkinson M. Woolrich M. W. Beckmann C. F. Behrens T. E. Johansen-Berg H. (2004). Advances in functional and structural MR image analysis and implementation as FSL. Neuroimage 23(Suppl. 1), S208–S219 10.1016/j.neuroimage.2004.07.051 15501092

[B31] Sutherland D. H. Olshen R. Cooper L. Woo S. L. (1980). The development of mature gait. J. Bone Joint Surg. Am. 62, 336–353 7364807

[B32] Timmons B. W. Leblanc A. G. Carson V. Connor Gorber S. Dillman C. Janssen I. (2012). Systematic review of physical activity and health in the early years (aged 0–4 years). Appl. Physiol. Nutr. Metab. 37, 773–792 10.1139/h2012-070 22765840

[B33] Wang L. Yu C. Chen H. Qin W. He Y. Fan F. (2010). Dynamic functional reorganization of the motor execution network after stroke. Brain 133, 1224–1238 10.1093/brain/awq043 20354002

[B34] Welker K. M. Patton A. (2012). Assessment of normal myelination with magnetic resonance imaging. Semin. Neurol. 32, 15–28 10.1055/s-0032-1306382 22422203

[B35] Williams H. Monsma E. (2006). Assessment of Gross Motor Development in Preschool Children. Hillsdale, NJ: Lawrence Erlbaum

[B36] Wu Y. Zhong Z. Lu M. He J. (2011). Statistical analysis of gait maturation in children based on probability density functions. Conf. Proc. IEEE Eng. Med. Biol. Soc. 2011, 1652–1655 10.1109/IEMBS.2011.6090476 22254641

[B37] Yamada K. (2009). Diffusion tensor tractography should be used with caution. Proc. Natl. Acad. Sci. U S A 106:E14 10.1073/pnas.0812352106 19179404 PMC2650181

[B38] Yeo S. S. Chang M. C. Kwon Y. H. Jung Y. J. Jang S. H. (2012a). Corticoreticular pathway in the human brain: diffusion tensor tractography study. Neurosci. Lett. 508, 9–12 10.1016/j.neulet.2011.11.030 22197953

[B39] Yeo S. S. Choi B. Y. Chang C. H. Kim S. H. Jung Y. J. Jang S. H. (2012b). Evidence of corticospinal tract injury at midbrain in patients with subarachnoid hemorrhage. Stroke 43, 2239–2241 10.1161/STROKEAHA.112.661116 22700530

[B40] Yeo S. S. Kim S. H. Jang S. H. (2013). Proximal weakness due to injury of the corticoreticular pathway in a patient with traumatic brain injury. NeuroRehabilitation 32, 665–669 10.3233/NRE-130889 23648621

[B41] York D. H. (1987). Review of descending motor pathways involved with transcranial stimulation. Neurosurgery 20, 70–73 10.1097/00006123-198701000-00021 3543726

